# Bioinspired and bristled microparticles for ultrasensitive pressure and strain sensors

**DOI:** 10.1038/s41467-018-07672-2

**Published:** 2018-12-04

**Authors:** Bing Yin, Xiaomeng Liu, Hongyan Gao, Tianda Fu, Jun Yao

**Affiliations:** 1 Department of Electrical and Computer Engineering, University of Massachusetts, Amherst, 01003 USA; 2 Institute for Applied Life Sciences, University of Massachusetts, Amherst, 01003 USA

## Abstract

Biological sensory organelles are often structurally optimized for high sensitivity. Tactile hairs or bristles are ubiquitous mechanosensory organelles in insects. The bristle features a tapering spine that not only serves as a lever arm to promote signal transduction, but also a clever design to protect it from mechanical breaking. A hierarchical distribution over the body further improves the signal detection from all directions. We mimic these features by using synthetic zinc oxide microparticles, each having spherically-distributed, high-aspect-ratio, and high-density nanostructured spines resembling biological bristles. Sensors based on thin films assembled from these microparticles achieve static-pressure detection down to 0.015 Pa, sensitivity up to 121 kPa^−1^, and a strain gauge factor >10^4^, showing supreme overall performance. Other properties including a robust cyclability >2000, fast response time ~7 ms, and low-temperature synthesis compatible to various integrations further indicate the potential of this sensor technology in applying to wearable technologies and human interfaces.

## Abstract (web summary)

The potential of electromechanical sensors has been limited by low volumetric density in sensing sites. Here, the authors demonstrate ultrasensitive pressure and strain sensors using ZnO microparticles that have high-aspect ratio and high-density nanostructured spines mimicking bristles in insects.

## Introduction

Electromechanical sensors have a long history and are becoming increasingly important for developing human interfaces for motion capture, health monitoring, and disease diagnostics^1–3^. They can also emulate the tactile receptors in human skin for developing electronic skin^4,5^. A progressive vision is to integrate sensor elements with computing devices to form smart, responsive, and intelligent systems for humanoid technologies and prosthetics^5,6^. This system-level mimicry is often implemented at component levels, which include using synaptic devices for signal processing^7^, skin-like materials for substrate engineering^8^, and bioinspired structures in sensors for improving signal transduction^9–14^.

Emulating biological structures for sensor design has a valid point. Mechanotransduction is ubiquitous and exists even in the simplest life forms of unicellular organisms^15,16^. It plays the central role in biological systems for their survival, interaction, and adaptation^15^. Consequently, these biological sensory organelles often have optimized structures that can attain high sensitivity and specificity. Borrowing from biological structures for sensor design can lead to functional imitation and improvement. For example, the introduction of cracks in a conductive thin film, which mimics the surface topology in a spider’s sensory system, has led to ultrasensitive strain sensors (e.g., gauge factor ~2000) that can track various human vibrations^9,10^. An alternative strategy is to emulate the geometries in sensory organelles to improve sensitivity and multifunctionality^11–14^. In a broad sense, as biological organelles have innate three-dimensional (3D) geometries, the common strategy of employing 3D structural engineering in the devices often infers a subconscious mimicry^17–19^.

One common type of sensory organelles are the bristles or tactile hairs ubiquitous in insects^20^. The bristle acts as an lever arm to translate mechanical stimuli into pressing force to the sensory dendrites at the base for bioelectrical signaling^15,20^. In particular, the tapering geometry or spine structure confers a clever design that not only promotes signal transduction for high sensitivity, but also protects the bristle from mechanical breaking^21,22^. Similar structural engineering by using vertical nanowires has been employed in mechanical sensors and yielded enhanced sensing performance^23–25^, although these efforts were limited in replicating the nanostructured details such as the innate tapering in biological bristles. Also, typical synthesis and fabrication only yielded nanowire forests distributed over a plane^23–26^, which lacks a hierarchical 3D distribution that may potentially extend the sensitivity. Extending the planar nanowire growth to a conformal coating on polydimethylsiloxane (PDMS) pillar arrays has led to interlocked interface with improved sensitivity (6.8 kPa^−1^) and detection limit (0.6 Pa)^27^, although the relative large size in the PDMS pillar (e.g., tens of micrometers vs. nanowire coating thickness <5 µm) has limited the contact volume to <10% of the total device volume and hence may have limited further improvement. Introducing nanostructured arms or protrusions on the surface of micro- and nanoparticles helped to promote a 3D spherical distribution in nanostructures for improved performance, yielding low detection limit (e.g., 0.3–1 Pa) and high sensitivity (e.g. 2–9 kPa^−1^) in pressure sensing^13,14^. Still, the limited aspect ratio (e.g., <5) or surface density (e.g., <20 per particle) in the protruding nanotructures^13,14^ may have prevented a further optimization in sensing performance.

## Results

### General concept

**Fig. 1:**
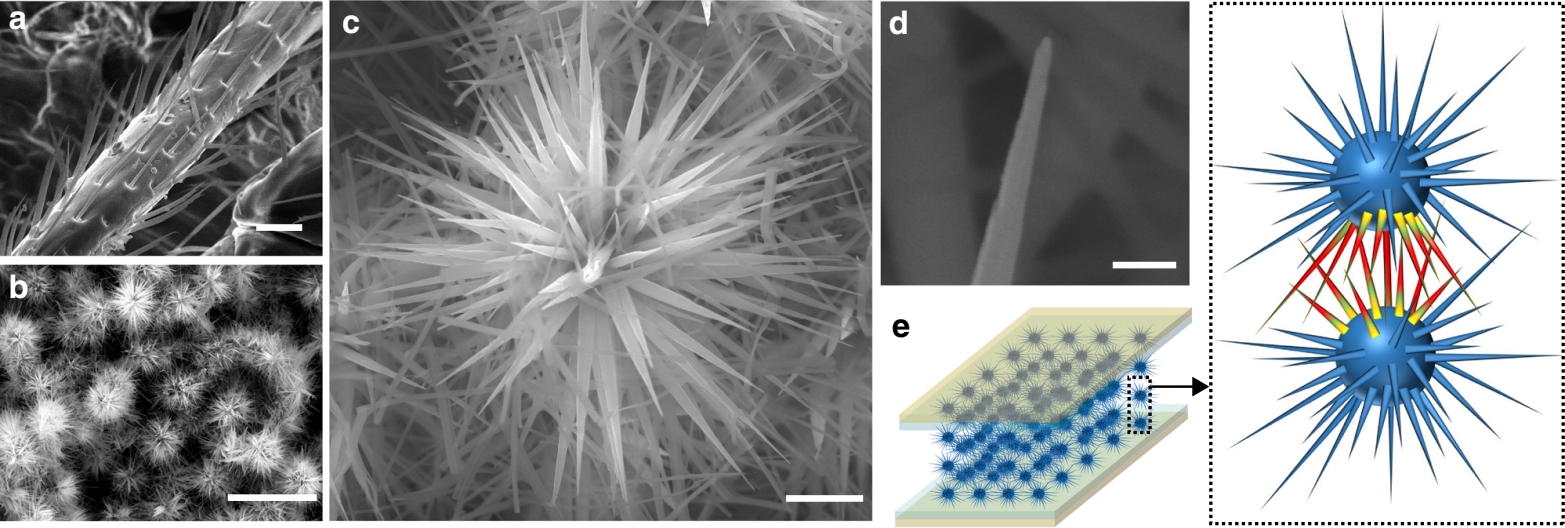
ZnO-microparticle morphology and sensor concept. **a** Scanning electron microscopy (SEM) image of a leg from an ant, showing the hierarchical distribution of tapering bristles. Scale bar, 20 µm. **b** SEM image of SUSMs packing together in a thin film. Scar bar, 10 µm. **c** High-magnification SEM image of one SUSM, showing a forest of nanostructured spines. Scale bar, 1 µm. **d** Zoom-in SEM image of the tip of a spine. Scale bar, 100 nm. **e** (Left panel) Schematic of a sensor device made from a SUSM thin film sandwiched between two electrodes. (Right panel) Schematic highlights the resistance modulation at local spine–spine sites induced by mechanical stimuli, with the color gradient indicating induced strain

To mimic the structure and function of bristles in insect (Fig. 1a), we used synthetic zinc oxide (ZnO) microparticles (Fig. 1b, c) that feature high-aspect-ratio (e.g., >20) and high-density (e.g., >150 per particle) nanostructured spines to construct mechanical sensors. The tapering geometry in the spines resembles that in biological bristles (Fig. 1d), and the overall morphology of the microparticle resembles that of a sea urchin. We term it as sea urchin-shaped microparticles (SUSMs). The high density and spherical distribution in the spines determine that mechanical stimuli can be collected from all directions. The high-aspect ratio and bristle-like geometry in the spines indicate that similar enhancement in signal transduction and mechanical resilience^21,22^ may be obtained in individual spines. Collectively, a thin film made from these SUSMs is expected to have a broad collection of local signal amplifications contributed from both resistive (e.g., inter-spine contacts) and piezoresistive (e.g., spine bending) effects (Fig. 1e), leading to improved sensitivity for both pressure and strain sensing. The constructed sensors feature a static-pressure detection down to 0.015 Pa, a pressure sensitivity up to 121 kPa^−1^, and a strain gauge factor >10^4^, showing supreme overall performance. Other properties including a robust cyclability >2000, a fast response time ~7 ms, and a low-temperature solution processing compatible to various integrations have further indicated the potential of this sensor technology in applying to wearable technologies and human interfaces.

### Material and device preparations

The SUSMs were synthesized through a solution process at the temperature of 40 °C (see Methods and Supplementary Figure 1a)^28,29^. The average size of SUSMs can be controlled by growth time. A saturation in growth happened at ~12 h, yielding an average size ~5.5 μm (Supplementary Figure 1b). The average number of nanostructured spines in one SUSM is ~160 (Fig. 1c), which is largely independent of the growth time (Supplementary Figure 1c). This number is more than an order of magnitude larger than that in another sea-urchin-shaped particle used for mechanical sensors^13^, hence can yield substantially higher density in sensing sites for improved sensitivity. The average tapering angle in the spines is ~2–3°. The tapering results in an aspect ratio ~20–30 and a tip size ~10–20 nm in the spines (Fig. 1d), which are also independent of growth time. These features indicate a dominant longitudinal growth along the *c*-axis after the initial nucleation^29^. The size saturation in SUSMs can be accounted for by a growth competition in the spine forest, during which a lateral confinement at the base eventually slows down the elongation due to the tapering geometry. These geometric features also indicate that saturated-grown SUSMs, with the saturated length in spines and still smallest size in spine tips, will likely have the optimized size for sensing performance. Consequently, we focused on using saturated-grown SUSMs to construct sensor devices.

**Fig. 2:**
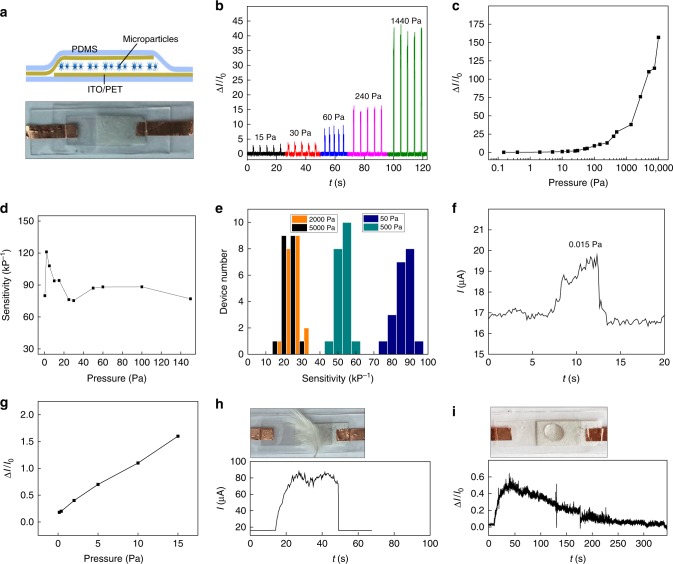
Pressure-sensing characterizations. **a** Schematic of the layered structure in the sensor (top panel) and photo of an actual fabricated device (bottom panel). **b** Current response (Δ*I*/*I*_0_) from a device applied with different pressures. The current was measured at a constant voltage bias of 5 V. **c** Current response from the device in the pressure range of 0.1–10^4^ Pa. **d** Device sensitivity, defined as the slope at the corresponding pressure, in the range of 0.1–150 Pa. **e** Statistical distribution of sensitivity at different pressures from 20 devices, with average sensitivity of 86.5 ± 4, 52.9 ± 3.7, 28 ± 3.4, and 23.4 ± 4.3 kPa^−1^ at pressures of 50, 500, 2000, and 5000 Pa, respectively. **f** Current response in a device at an applied static pressure of 0.015 Pa, and **g** its current response at the low-pressure range of 0.1–15 Pa, showing approximately linear relationship with an average slope ~94 kPa^−1^. **h** Current response in a device responding to a landed feather on top (top). **i** Current response in a device registering the dynamic evaporation process of a droplet of ethanol (~40 µL) on top of the device (top)

We used drop casting to assemble SUSM thin films, which is expected to yield self-organized packing in SUSMs for optimal electrical and mechanical properties. The film thickness was controlled by the solution volume over unit area, with the shape and size in the area controlled by a PDMS mold (Methods). The film (~1 × 2 cm^2^) was initially assembled and patterned on a polyethylene terephthalate (PET) substrate coated with ~130 nm indium-tin oxide (ITO) layer serving as the bottom electrode, and then capped with another PET/ITO layer as the top electrode (Fig. 2a). The device was further encapsulated by top and bottom PDMS layers (~1 mm thick) to maintain its structural integrity and stability during mechanical testing^30^. The current–voltage (*I–V*) response from the device features typical nonlinear behavior (Supplementary Figure 2). The non-linearity indicates a hopping conduction that is highly sensitive to inter-site distance and hence mechanical perturbation, which has been widely employed in sensor engineering for improved sensitivity^31,32^. Here it indicates a percolative conduction contributed from both an unintentional doping in ZnO material^33,34^ and inter-spine contacts, which can be favorable for achieving high sensitivity. Our initial test showed supportive evidence to above hypothesis, during which distinct and large current changes in the device were observed in response to different applied pressures (Fig. 2b). The retained signal amplitude with repetitive pressure loading also indicated the potential mechanical stability in the device.

### Pressure-response characterizations

As the percolative conduction is relevant to the average number of inter-spine contacts along the vertical path, film thickness is expected to affect the sensing performance. We found that film with a thickness ~70 µm yielded higher sensitivity compared to thinner or thicker ones (Supplementary Figure 3). Qualitatively, this thickness-dependent optimization can be explained by a competing effect in resistance distribution. For example, an initial increase in film thickness increases the contribution from inter-spine resistance (*vs*. material resistance and film-electrode interfacial resistance). As inter-spine resistance is expected to be most sensitive to mechanical perturbation (Fig. 1e, right panel), it will lead to an improved sensitivity. A further increase in film thickness, for a fixed bias voltage (e.g., 5 V) across the film, leads to a proportional reduction in the average voltage drop across individual inter-spine contacts. As a nonlinear hopping conduction assumes higher resistance at lower voltage bias^35^, the resultant higher resistance state (*R*_0_) will depress a relative resistance change (*ΔR*/*R*_0_) or pressure sensitivity. Figure 2c shows the pressure response in the device with optimized film thickness (~70 µm) in the pressure range of 0–10 kPa, showing unidirectional increase in current response (Δ*I*/*I*_0_) with the increase in applied pressure. A signal >1000% was achieved for pressure above 100 Pa. Meanwhile, a sensitivity of 75–121 kPa^−1^ was achieved in the range 0–200 Pa (Fig. 2d), which is more than an order of magnitude larger than values in previous particle-based sensors^13,14^ and among the highest in all pressure sensors^1,36,37^. The sensitivity maintains a value >15 kPa^−1^ in the broad pressure range of 200–10,000 Pa (Supplementary Figure 3b), which is distinctly different from previous systems where the sensitivity drops drastically with increasing pressure^13,18,19,36^. This sensitivity in the medium pressure range is more than an order of magnitude higher than that in a system initially having similarly high sensitivity at low-pressure range (e.g., <30 Pa)^36^. Importantly, statistical data from 20 devices shows narrow distributions of the sensitivity across different pressures (Fig. 2e), further indicating the uniform and reproducible performance.

We further tested the pressure response in the low-pressure region (e.g., <20 Pa) to determine the sensing limit in the device. Specifically, a force detection limit ~3 µN, corresponding to a pressure of ~0.015 Pa, was resolved with a signal-to-noise ratio >7 (Fig. 2f). To our knowledge, this is the lowest static pressure detected among developed mechanical sensors^1,37^. The pressure response in the low-pressure region was approximately linear (Fig. 2g), with an average sensitivity ~94 kPa^−1^. The high sensitivity in the device has enabled the detection of minute mechanical events such as the landing of a feather (Fig. 2h), which still yielded a signal change >300%. The high resolution in static-pressure sensing, as opposed to dynamic-pressure sensing that only registered the onset and offset of mechanical events^38,39^, can also provide real-time tracking of small dynamics. Figure 2i shows the response from the evaporation of a droplet of ethanol (~40 µL) on top of the device. For the linear response in the low-pressure range (Fig. 2g), change in the sensing signal can be linearly related to the mass change in the ethanol droplet. For a droplet with small contact angle (e.g., <40°), the evaporation rate is approximately constant^40^. Consequently, an approximately linear decrease in ethanol mass and hence sensing signal is expected, which is consistent with the response curve (Fig. 2i). The estimated evaporation rate, based on a radius ~0.3 cm in the droplet, was ~1.2 mmol s^−1^ m^−2^, which is close to reported experimental value^41^. As control, a water droplet, which has a much slower evaporation rate, produced a plateau in the sensing signal (Supplementary Figure 4). These results indicate that the device may be used to study the evaporation dynamics in various liquid droplets^40^.

### Mechanical-cyclability characterizations

**Fig. 3:**
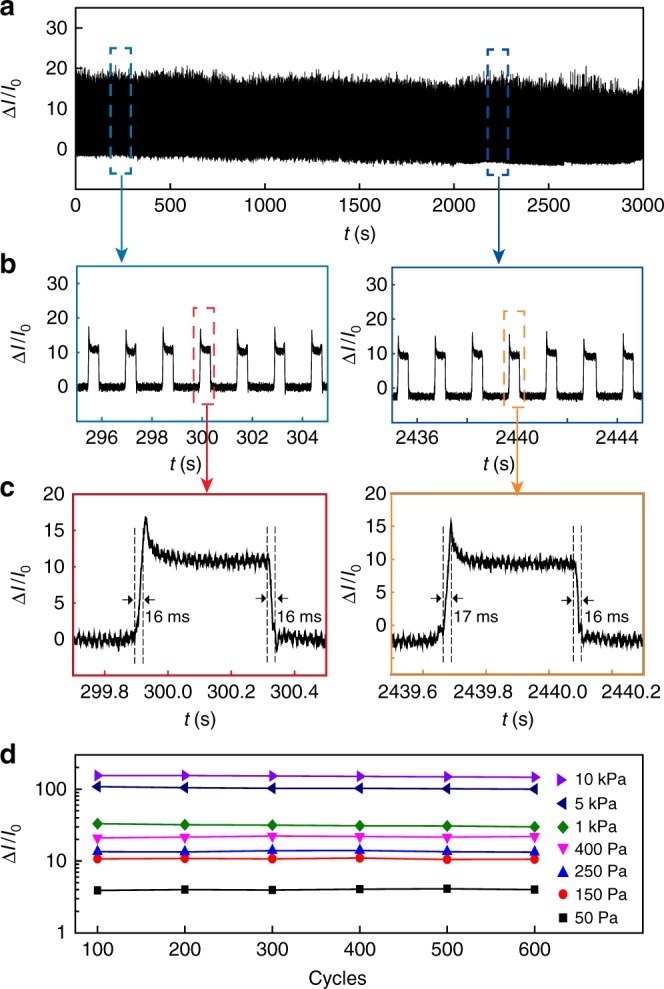
Mechanical cyclability. **a** Current response from a device during a continuous, repetitive (e.g., >2000 cycles) pressure loading (150 Pa, *T* = 1.5 s, duty cycle 34%). **b** Zoom-in signals at the beginning (left panel) and final (right panel) stages of the cycling test, showing that the signal amplitude ~11 was well maintained. **c** Zoom-in signals in one cycle at the beginning (left panel) and final (right panel) stages of the cycling test, showing that a response time ~16 ms was maintained. **d** Signal amplitudes at different applied pressures during cycling tests

We also tested the reliability of the sensor during repetitive pressure loading. The amplitude in the sensing signal, with an initial value ~11, showed little change after >2000 cycles (Fig. 3a, b). The response time (~16 ms) was also well maintained throughout the cycles (Fig. 3c). It should be noted that this response time was not intrinsic to the device and largely limited by the pressure loading rate (e.g., ~1.5 mm/s). Independent test using faster loading rate resulted in a faster response time ~7 ms (Supplementary Figure 5), which is among the lowest values in typical mechanical sensors^1,4^. Cycling at different pressures have yielded similar trends in maintaining the performance (Fig. 3d). Collectively, the robust durability in the device indicates that the intertwined spine–spine interactions, protected from mechanical breaking by the biomimetic tapering geometry^21,22^, have created an effective cushioning mechanism for reproducible sensing. The fast response and robust cyclability in the device coincide with the observation that a continuous loading–unloading process yielded little hysteresis in the sensing response (Supplementary Figure 6). All these results further indicate the absence of viscoelastic behavior that can generally degrade device performance^27^.

### Strain response

**Fig. 4:**
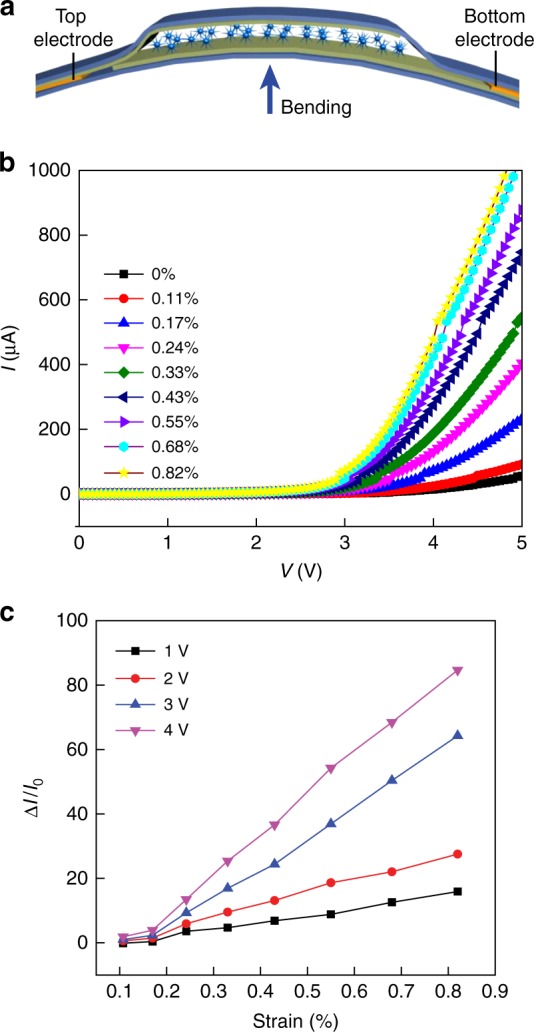
Strain response in the sensor. **a** Cross-section schematic of the bending configuration in the device. **b** *IV* curves in the device at different bending strains. **c** Current responses in the device at different bending strains, recorded at different bias voltages (1–4 V)

The strain response in the device was tested by bending the device along central line (Fig. 4a). Figure 4b shows the *IV* responses at different strain levels, during which a continuous increase in current was observed with increasing strain. Figure 4c shows the current response with respect to strain change at different biases, showing approximately linear relationships. As analyzed previously, a larger voltage bias (e.g., 4 V) tends to produce a larger response or sensitivity in the device, due to a reduced *R*_0_ and hence improved Δ*R*/*R*_0_ in a nonlinear hopping conduction^35^. In this case, the gauge factor is estimated to be >10^4^, which is the highest value reported and orders of magnitude larger than values in typical strain sensors^1,42^. This ultrahigh sensitivity in strain sensing is consistent with the ultrahigh sensitivity in pressure sensing, as the bending process effectively produces normal stress in the film^43^.

### Wearable device demonstration

**Fig. 5:**
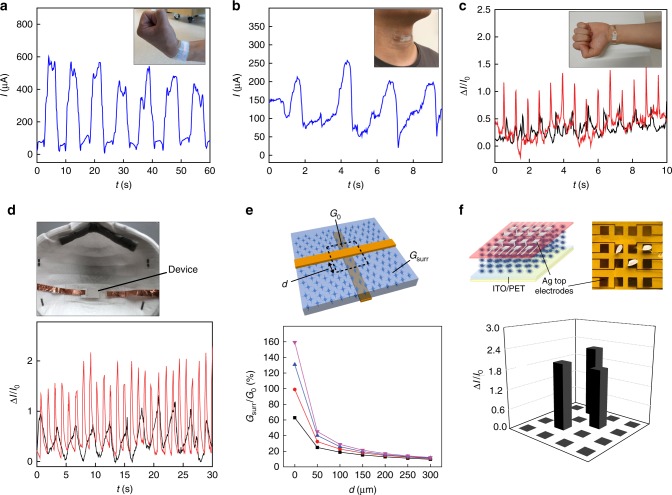
Wearable demonstration and device integration. **a** Sensing signals from a prototype device interfaced with the wrist (inset) for tracking the bending movement. **b** Signals from the device interfaced with the neck (inset) for tracking the swallowing motion. The increased baseline current in **a**, **b** compared to that in a free device (Supplementary Figure 2c) resulted from a static/baseline pressure exerted on the device for maintaining a close interface. **c** Registered pulse signals from the wrist, with the black curve indicating a normal heart rate (~66 beats per minute) and red curve the rate (~96 beats per minute) after exercise. **d** Respiratory signals (bottom panel) registered by a device placed in a mask (top panel), with the black curve indicating a normal rate (~21 breaths per minute) and red curve the rate (~52 breaths per minute) after exercise. **e** Simulated conductance distribution (bottom panel) in a continuous SUSM thin film sandwiched between two crossed top and bottom electrodes (top panel). *G*_0_ denotes the conductance contributed from the squared region (dashed lines) centered at the cross-point. *G*_surr_ denotes the conductance contributed from the rest area that has a distance *d* away from the edge of electrode. The widths of both electrodes are 100 µm. The purple, blue, red, and blue curves correspond to SUSM film thicknesses of 30, 50, 70, and 90 µm, respectively. **f** (Top left) Schematic of a tactile sensor array made from a continuous SUSM film (70 µm thick), featuring individually addressable 4 × 4 top (Ag) electrodes and a common bottom (ITO) electrode. (Top right) Actual photo of fabricated 4 × 4 device array; each device size is 0.5 × 0.5 cm^2^ and the pitch size is 0.9 cm. (Bottom) Current responses from all the devices corresponding to local pressure exerted by three grains of rice

The high sensitivity in both pressure and strain sensing indicates that the device can be a good candidate for wearable technology tracking human mechanical signals. Prototype devices were used to interface different body parts for mechanical detections. These demonstrations included capturing the bending movement in the wrist (Fig. 5a) that can be used for remote control^2^, detecting swallowing motion from the neck (Fig. 5b) that is relevant to voice recognition^44^, and monitoring physiological pulse (Fig. 5c) for health diagnostics. Note that the device size used for these interfaces was still kept at 1 × 2 cm^2^. As the pulse pressure from radial artery is quite local (e.g., width ~0.2 cm), the effective pressure exerted on the device or the sensing signal is estimated to be 10× reduced, which was commonly observed in other wearable pulse monitors^14,37,45^. Nevertheless, due to the ultrahigh sensitivity in the device, without interfacial engineering^2,3^, an average signal >100% was routinely obtained, which is larger than typical values from wearable mechanical sensors^1,18,35^. Evidently, a reduction in device length to half of its initial value (e.g., 1 cm) has yielded a ~2× improvement in signal (Supplementary Figure 7). The device can also detect pressure exerted by air from respiration, hence enabling respiratory monitoring (Fig. 5d). The high sensitivity in the devices has enabled not only the tracking of signal frequencies, but also registration of the changes in amplitudes corresponding to different physical states (red curves, Fig. 5c, d).

The low-temperature solution synthesis indicates that a large-scale integration of SUSMs on various polymeric flexible substrates is feasible for broad applications. Our method by using PDMS mold can yield well defined and isolated SUSM patterns for device definition (Methods). Lithographic definition of PDMS mold^46,47^, combined with drop casting, can lead to scalable device integration. Post-coating processing such as masked etching^48^ can also lead to isolated patterns for defining individual devices. An attractive alternative, nevertheless, is to use a continuous thin film for local signal differentiation by utilizing an anisotropic conduction between the vertical and horizontal directions, which can be advantageous for reducing post-processing cost and contamination. One such application is for addressable tactile sensing or pressure mapping^4,5^. We evaluated the feasibility of using a continuous SUSM film for pressure mapping with crossbar architecture^30^, in which two crossed top and bottom electrodes are used to address the local pressure response in the overlapping area (Fig. 5e, top panel). We performed simulation (Supplementary Figure 8) to reveal the conductance contribution from surrounding area (*G*_surr_) compared to that from the local area (*G*_0_). Line width of 100 µm was used for both top and bottom electrodes, yielding a crossing area ~100 × 100 µm^2^ close to the fine tactile resolution in human^4^. Fig. 5e (bottom panel) shows the conductance contribution (*G*_surr_/*G*_0_) with respect to the distance *d* away from the electrode edge. The result shows that for a distance *d* > 100 µm, the overall conductance contribution from surrounding area is <20% in a 30 µm thick film. This indicates that a continuous thin film can still achieve spatial resolution (e.g., ~300 µm) close to that in human touch^4^ without the risk of false sensing contributed from neighboring area (for the eventual implementation of crossbar architecture, we assume rectifying element will be added to each cross-point to avoid the sneak path problem^49^, and this is relatively easy in ZnO materials as a rectifying effect can be readily obtained by the contacts^50^). We made a proof-of-concept demonstration of pressure mapping using a 4 × 4 sensor matrix made from a continuous SUSM film with a shared common bottom electrode (Fig. 5f, top panel). The result shows that each device element can not only resolve small pressure (e.g., a grain of rice ~30 mg or 0.3 mN), but also effectively decouple from each other (Fig. 5f, bottom panel).

## Discussion

We have demonstrated a bioinspired mechanical sensor by mimicking both the fine structure (e.g., nanostructured tapering) and general organization (e.g., global hierarchical distribution) of bristles in insect. Such comprehensive mimicry underlies the supreme overall performance featuring ultrahigh sensitivity in both pressure and strain sensing. The concept and methodology may be adopted to further tailor sensor functionality: for example, filtrating the SUSM scaffold with polymeric elastomer may lead to ultra-flexible or stretchable sensors. The low-temperature solution synthesis also opens the possibility of using printing method to write SUSM patterns for printed electronics^51^, although details in rheology and thermal effect for accelerating synthesis in droplet environment need to be further explored.

## Methods

### Synthesis of ZnO SUSMs

The ZnO microparticles were synthesized using a low-temperature solution process^28,29^. Specifically, two solutions, with 6.4 g sodium hydroxide (NaOH; Fisher Scientific) dissolved in 40 ml deionized (DI) water and 3.6 g zinc acetate dihydrate [Zn(CH_3_COOH)2•2H_2_O); Fisher Scientific] in 40 ml DI water, were first prepared. Then, the zinc acetate aqueous solution was added into the NaOH solution, followed by thorough stirring to form a transparent solution. The obtained solution was transferred to a Teflon-sealed autoclave and kept at 40 °C for 1–24 h. During the process, an overall reaction took place following^29^ Zn^2+^ + 4OH^−^ → [Zn(OH)4]^2−^ and [Zn(OH)_4_]^2−^ → ZnO + H_2_O + 2OH^−^. At the early stage (e.g., ~5 min), the transparent solution gradually became slightly turbid, indicating the formation of primary nuclei (Supplementary Figure 1a). The formed crystal nuclei subsequently grew to form initially faceted nanostructures. Due to the wurtzite structure in ZnO, the facets grew along the *c*-axis to elongate and form nanostructured spines. After ~1 h, a complete sea-urchin-shaped structure was formed (Supplementary Figure 1c). Further increasing reaction time would increase the lengths in the nanostructured spines, with a saturation taking place ~12 h. Finally, the formed microparticles were collected (e.g., through precipitation on a glass substrate), thoroughly washed with DI water, and dried in air at 90 °C.

### Device fabrication

The sensor devices were constructed by sandwiching a thin film of ZnO SUSMs between two ITO-coated PET films (127 µm thick, 60 Ohm/□; MilliporeSigma). Briefly, two PET films (1 × 2 cm^2^) were subsequently cleaned in acetone, ethanol, and DI water in an ultrasonic bath. A copper tape (2.5 mm wide) serving as extending electrode was bonded to each PET film. A 1 mm thick PDMS film (Sylgard 184, 10:1 mix ratio; Dow Corning) was cured, cut out an ~1 × 2 cm^2^ opening, and covered on one PET film for patterning SUSM film. ZnO SUSMs (~200 mg) were dispersed in 20 mL ethanol in an ultrasonic bath. Prepared SUSM solution (~100 μL) was drop casted in the PDMS opening and dried at 60 °C, yielding ~35 µm thick film. Multiple drop castings lead to proportional increase in film thickness. The PDMS mold was peeled off after the assembly. A second ITO-coated PET film was placed on top of the prepared SUSM film to serve as the top electrode. The fabricated device was sandwiched between two plasma-treated (10 W, 3 min) PDMS films (2 × 10 cm^2^, 1 mm thick) to maintain the mechanical stability during electromechanical testing. Prototype devices were taped to body parts for mechanical signal detections. For device arrays, a 5 × 5 cm^2^ ZnO SUSM film (70 µm thick) was deposited on the bottom ITO-coated PET film following the same procedure. Then, a 4 × 4 Ag electrode array (each electrode size ~0.5 × 0.5 cm^2^, pitch size 0.9 cm) was printed on a flexible Polyimide (PI) film (25.4 μm thick; Kaneka Apical) using a desktop inkjet printer (DMP-2831; Dimatix Corp.), followed by sintering at 230 °C for 3 min. The PI film was then placed on top of the SUSM film serving as addressable top electrodes.

### Characterizations

The ZnO SUSMs were imaged by a field emission scanning electron microscope (FESEM, Magellan 400). The morphology of bristles on the insect’s leg were imaged by a tabletop scanning electron microscope (EM-30; Element PI Inc.). Electrical transport properties or *IV* curves of the device were measured using a Keithley 4200 Parameter Analyzer. For electromechanical testing, the pressures were applied using a mechanical testing stage (ESM303; Mark-10 Inc.) equipped with a force gauge (M7-5; Mark-10 Inc.) and computerized control system. The mechanical bending in film was performed using a home-built bending stage. The current responses in the devices were amplified with a preamplifier (DL-1211; DL Instruments) and the output data were recorded at an acquisition rate of 1 kHz using a 16-channel A/D converter (Digidata 1440 A; Molecular Devices) interfaced with a computer running pClamp 11 electrophysiology software (Molecular Devices; Axon Laboratory).

## Electronic supplementary material

Supplementary Information

## Data Availability

The data that support the findings of this study are available from the corresponding author upon reasonable request.
