# Supplementary material for: Bioinspired and bristled microparticles for ultrasensitive pressure and strain sensors
Source: Nat Commun. 2018 Dec 4;9:5161. doi: 10.1038/s41467-018-07672-2 (PMC6279775; doi:10.1038/s41467-018-07672-2)
Supplement: Supplementary file 1 — Supplementary Information [file 41467_2018_7672_MOESM1_ESM.pdf]

Supplementary Information

Bioinspired and Bristled Microparticles for Ultrasensitive Pressure and Strain Sensors

B. Yin *et al.*

This file includes:

Supplementary Figures 1–8
Supplementary References 1–2

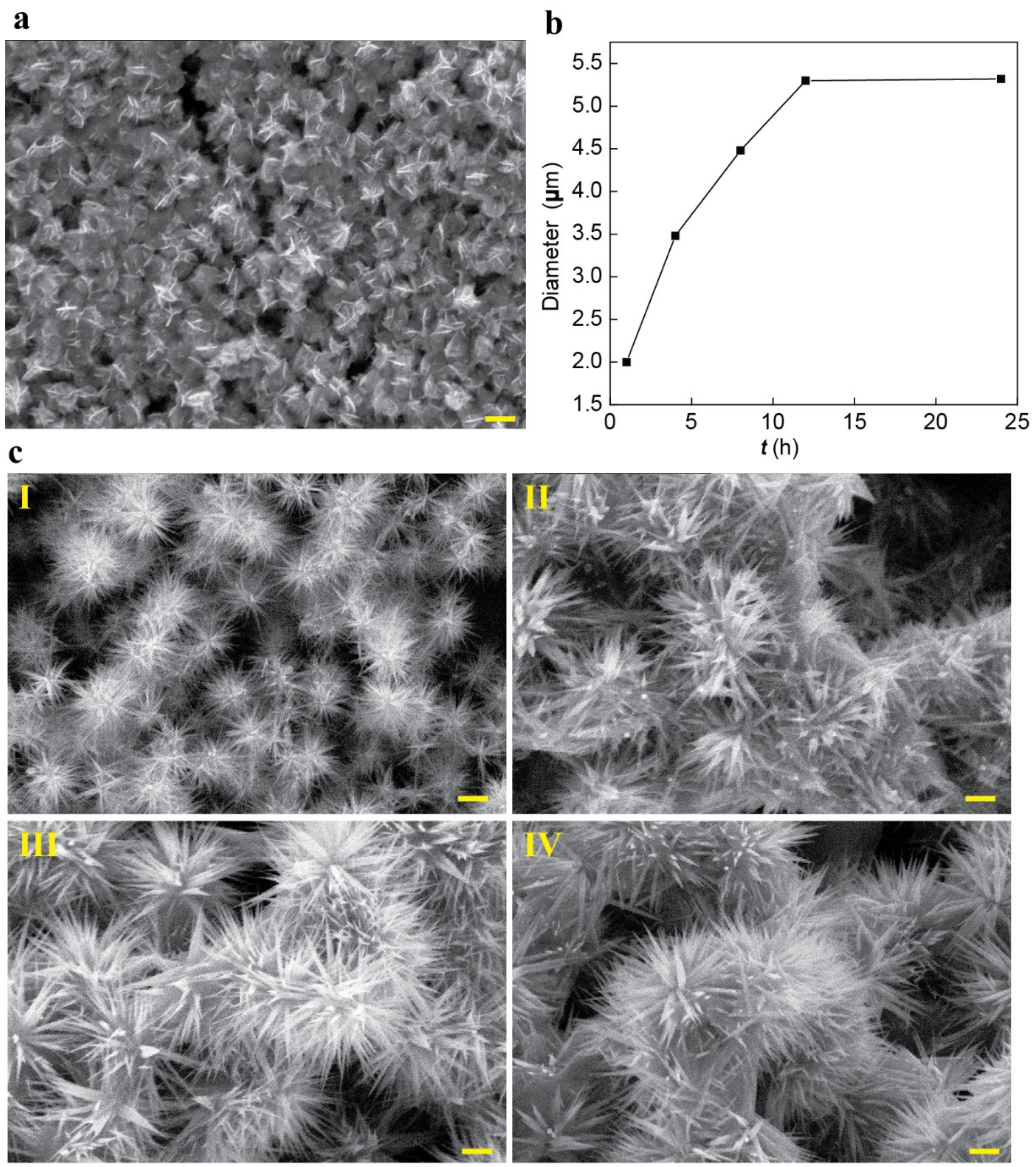

Supplementary Figure 1. ZnO SUSM growth trend. **a**, Faceted crystal nuclei formed at the early stage of reaction (~5 min) in the solution. After 1 h, sea urchin-shaped nanostructures were formed. Scar bar, 1 μm. **b**, Average diameters in ZnO SUSMs with respect to growth time. **c**, SEM images of SUSM morphologies at different growth time, ranging from 1 h (I), 4 h (II), 8 h (III) to 24 h (IV). Scale bars, 1 μm.

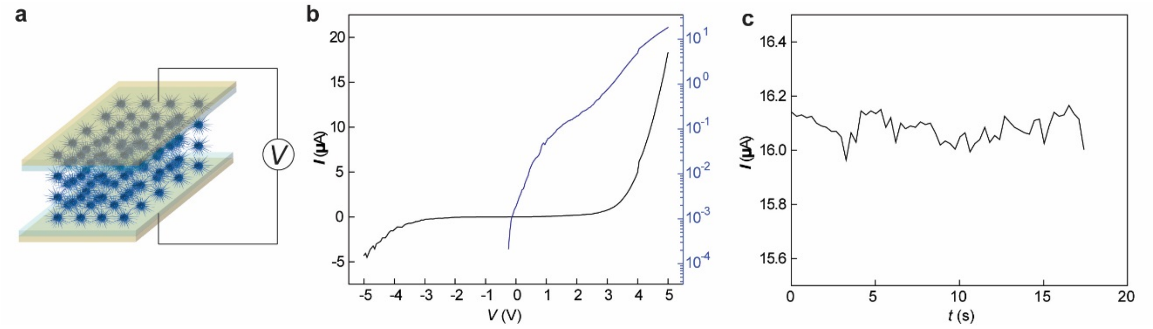

Supplementary Figure 2. **a,** Schematic of device with bias voltage applied between the top and bottom ITO/PET electrodes. **b**, Current-voltage (*IV*) response from a typical device, showing the non-linear conduction. The black curve corresponds to the linear Y-axis on the left, and the blue curve is the same *IV* shown in a logarithmic Y-axis (right). **c**. The typical current baseline from a device biased at 5 V, with a noise level ~0.2 μA. All the pressure sensing characterizations in the devices used the same voltage bias, unless otherwise specified.

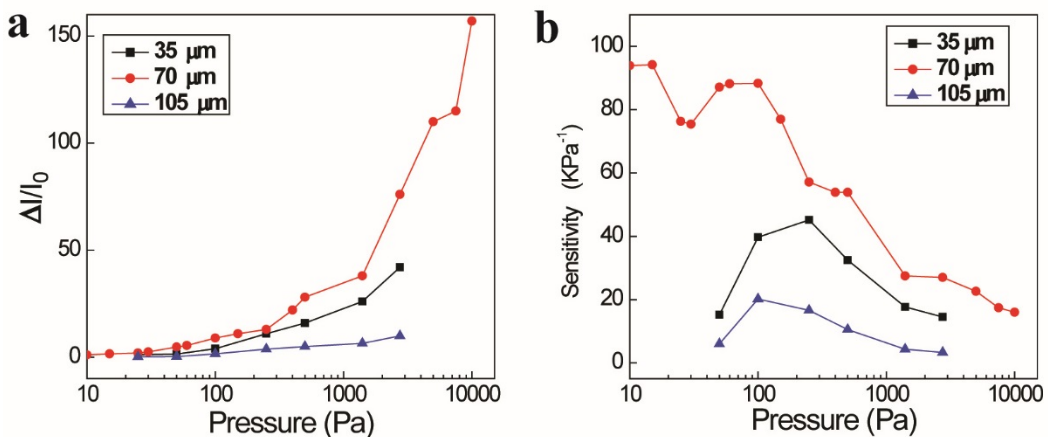

Supplementary Figure 3. **a**, Pressure responses in devices with different SUMS film thicknesses. **b**, Corresponding sensitivity at given pressure in the devices. The data shows that a 70 µm film thickness (red curve) yielded optimal performance compared to thinner (35 µm, black curve) and thicker (105 µm, blue curve) films.

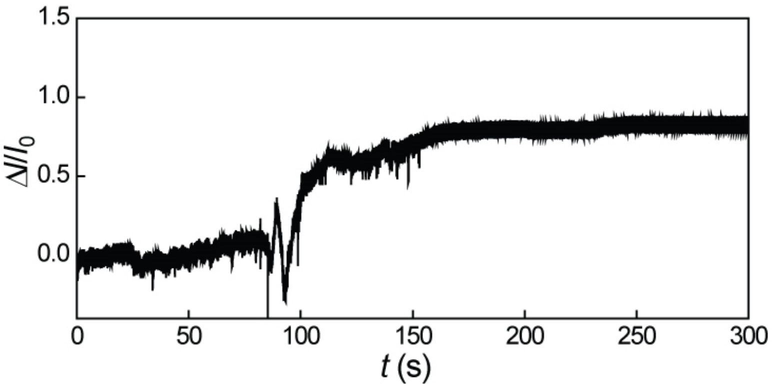

Supplementary Figure 4. Current response in a device registering the landing of a droplet of water (~40 µL) on top of the device.

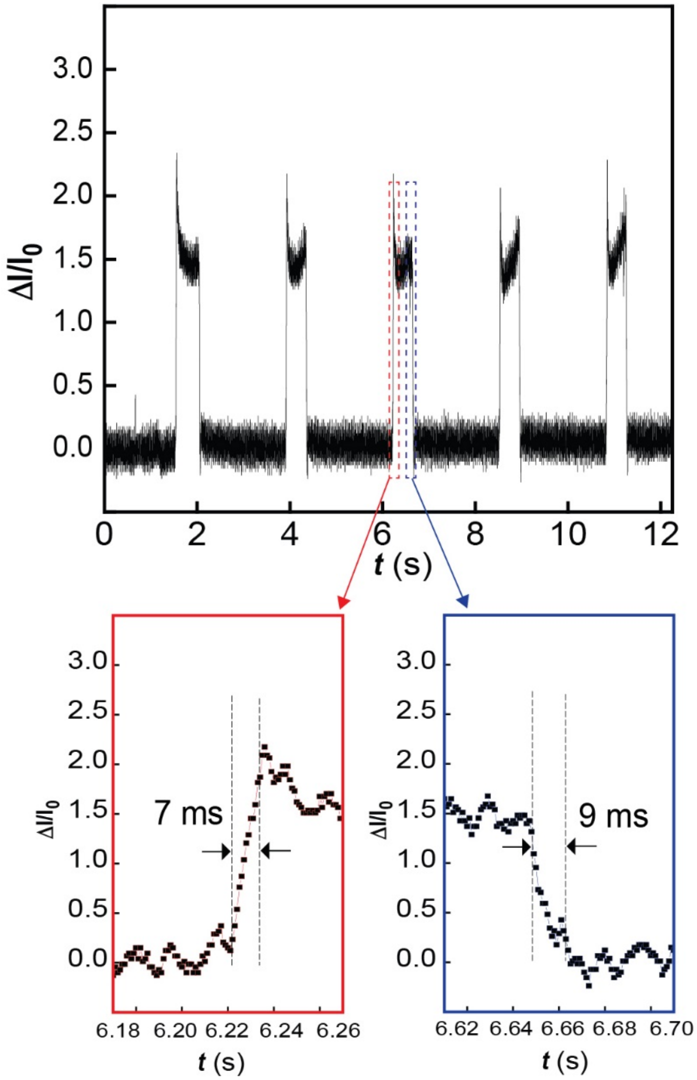

Supplementary Figure 5. Response time in a device with faster loading rate (2 mm/s, 15 Pa), showing sub-10 ms responding time.

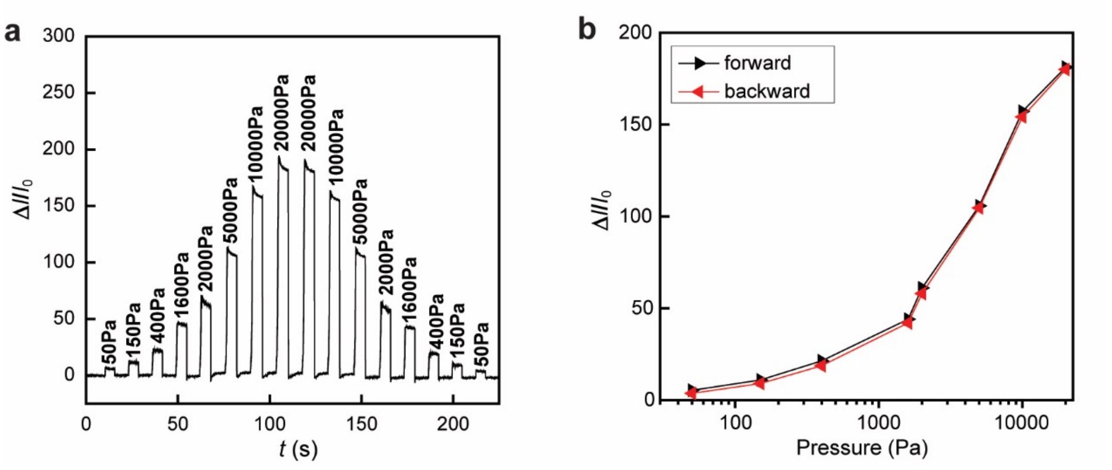

Supplementary Figure 6. **a,** Current signal in a device during continuous pressure loading/unloading. **b,** Corresponding data points during the foreward and backward sweeps, showing reproducible response with little hysteresis.

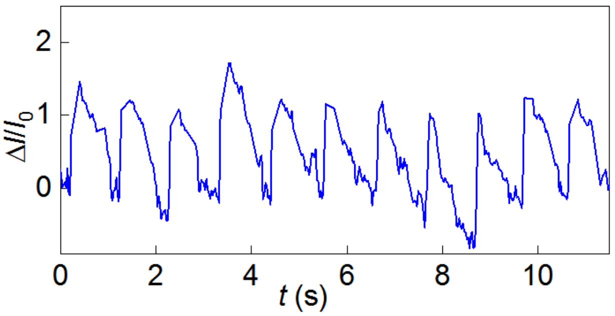

Supplementary Figure 7. Pulse signal measured by using a device with half the length (~1 cm), showing ~2× improvement in the sensing signal from normal heart rate (compared to black curve in Fig. 5c in the main text).

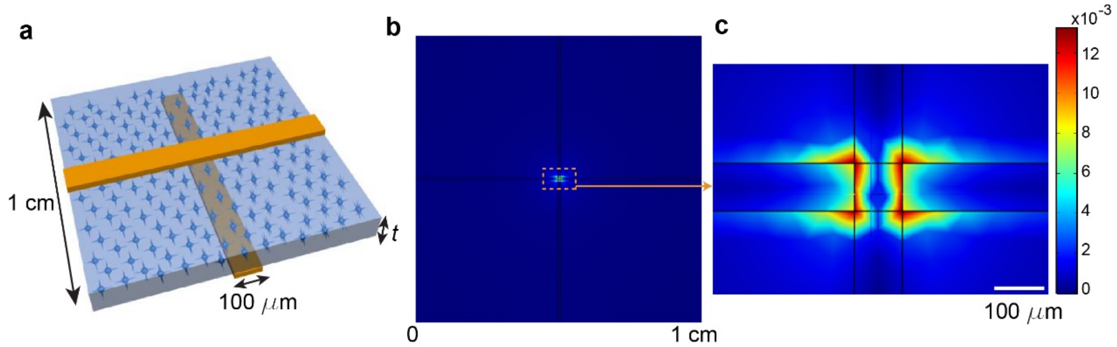

Supplementary Figure 8. Conductance distribution in a crossbar device by simulation. The simulation was done by using the finite element method (COMSOL 4.4). **a**, Schematic of the device configuration. The widths of both top and bottom electrodes were chosen to be 100 μm^2, which is close to the fine resolution in human touch.[1] The thickness (*t*) in SUSM film was varied between 30-90 µm, which was experimentally shown to yield optical sensing performance (Supplementary Figure 3). The size of the continuous SUMS film was chosen to be 1×1 cm^2, yielding an aspect ratio (with respect to film thickness *t*) >100 for accurate convergence of the total conductance across the two crossed electrodes. An isotropic electrical conductivity (*e.g.*, 1×10^{-6} S/cm) was assumed[2] (eventually the actual value was not important as we were calculating the ratio in conductance contribution). Since the electrical conductivity in the Au electrode (*e.g.*, 4×10^{5} S/cm) is orders of magnitude larger than that in ZnO, both top and bottom electrodes were assumed to be ideal conductors. The top electrode ($10^2\times10^4$ μm^2, W×L) was applied with a +5 V voltage bias used in our experimental tests, and the bottom electrode ($10^2\times10^4$ μm^2, W×L) was grounded. The current distribution in the ZnO SUSM film was calculated based on charge-density equation ($\nabla \cdot J = Q_{j,\varphi}$), Ohm's law ($J = \sigma E + J_e$), and electrostatic equation ($E = -\nabla\varphi$), where J is current density, Q is charge density, E is electric field, σ is electrical conductivity, and φ is the electric potential distribution. **b**, Counter map of current distribution (top view) over the entire thin film. **c**, Local current density (A/m^2) in the central region near the crossing point.

Supplementary References

1. Hammock, M. L. et al. The evolution of electronic skin (E-Skin): a brief history, design considerations, and recent progress. *Adv. Mater*. **25**, 5997-6038 (2013).
2. Caglar, J., Ilican, S., Caglar, Y. & Yakuphanoglu, F. Electrical conductivity and optical properties of ZnO nanostructured thin film. *Appl. Surf. Sci*. **255**, 4491-4496 (2009).
